# The utility of clinical decision tools for diagnosing osteoporosis in postmenopausal women with rheumatoid arthritis

**DOI:** 10.1186/1471-2474-9-13

**Published:** 2008-01-29

**Authors:** Caroline Brand, Adrian Lowe, Stephen Hall

**Affiliations:** 1 Clinical Epidemiology and Health Service Evaluation Unit, Melbourne Health, The Royal Melbourne Hospital – Royal Park Campus, Park House, Building 22, Parkville Vic 3052, Australia; 2 Department of Rheumatology, Royal Melbourne Hospital, Melbourne Health, The Royal Melbourne Hospital – Royal Park Campus, Park House, Building 22, Parkville Victoria 3052, Australia; 3 The Centre for Research Excellence in Patient Safety, Monash University, Victoria 3800, Australia; 4 Department of Rheumatology, Cabrini Hospital Malvern, Malvern Victoria 3144, Australia

## Abstract

**Background:**

Patients with rheumatoid arthritis have a higher risk of low bone mineral density than normal age matched populations. There is limited evidence to support cost effectiveness of population screening in rheumatoid arthritis and case finding strategies have been proposed as a means to increase cost effectiveness of diagnostic screening for osteoporosis. This study aimed to assess the performance attributes of generic and rheumatoid arthritis specific clinical decision tools for diagnosing osteoporosis in a postmenopausal population with rheumatoid arthritis who attend ambulatory specialist rheumatology clinics.

**Methods:**

A cross-sectional study of 127 ambulatory post-menopausal women with rheumatoid arthritis was performed. Patients currently receiving or who had previously received bone active therapy were excluded. Eligible women underwent clinical assessment and dual-energy-xray absorptiometry (DXA) bone mineral density assessment.

Clinical decision tools, including those specific for rheumatoid arthritis, were compared to seven generic post-menopausal tools to predict osteoporosis (defined as T score < -2.5). Sensitivity, specificity, positive predictive and negative predictive values and area under the curve were assessed. The diagnostic attributes of the clinical decision tools were compared by examination of the area under the receiver-operator-curve.

**Results:**

One hundred and twenty seven women participated. The median age was 62 (IQR 56–71) years. Median disease duration was 108 (60–168) months. Seventy two (57%) women had no record of a previous DXA examination. Eighty (63%) women had T scores at femoral neck or lumbar spine less than -1. The area under the ROC curve for clinical decision tool prediction of T score <-2.5 varied between 0.63 and 0.76. The rheumatoid arthritis specific decision tools did not perform better than generic tools, however, the National Osteoporosis Foundation score could potentially reduce the number of unnecessary DXA tests by approximately 45% in this population.

**Conclusion:**

There was limited utility of clinical decision tools for predicting osteoporosis in this patient population. Fracture prediction tools that include risk factors independent of BMD are needed.

## Background

Worldwide, the burden of osteoporotic fractures accounts for 0.83% of the global burden of non communicable disease [1] that can be measured in any community by associated morbidity, mortality and cost to the community [2]. Cost effective interventions are recommended for those with prevalent fragility fractures, however the role of diagnostic screening and intervention for those with low bone density and osteoporosis but no history of fracture is less well established. Low bone mass, most commonly measured using dual-energy xray absorptiometry (DXA) assessment of bone mineral density (BMD) is the most important risk factor for fracture [3] however other risk factors independent of BMD need to be considered and a number of these risk factors have been incorporated into targeted case finding clinical decision tools in attempts to improve the cost effectiveness of screening and potentially reduce the numbers of people undergoing unnecessary DXA tests [4-8]. The development of multiple tools may reflect disappointing performance of these tools when applied to populations other than the one within which they were developed [5].

Rheumatoid arthritis (RA) is an inflammatory arthritis that has been shown to be associated with low BMD, and a higher risk of fragility fracture [9,10]. Current guidelines advocate a case-finding approach to screening [11], with secondary causes such as rheumatoid arthritis being an indication. In Australia, diagnostic screening for osteoporosis in people with rheumatoid arthritis is subsidised by the National Health Scheme (Medicare) however a previous audit of assessment of osteoporosis risk by rheumatologists suggested use of DXA screening was limited to a subset of rheumatoid arthritis patients, primarily women who were on glucocorticosteroid treatment [12]. Whilst there is some evidence to support cost-effectiveness of screening and treating post menopausal women with rheumatoid arthritis who are starting corticosteroid treatment [13], the cost effectiveness of screening an unselected post-menopausal rheumatoid arthritis population has not been reported. Clinical criteria for case targeted BMD screening for rheumatoid arthritis patients are available [14] however their performance attributes have also varied across rheumatoid arthritis populations and it is uncertain whether they offer significant advantage over existing generic models for post-menopausal populations [15].

This study aimed to test the performance of generic and rheumatoid arthritis specific case finding clinical decision tools in a post-menopausal group of women with rheumatoid arthritis.

## Methods

A cross-sectional study of post menopausal women with rheumatoid arthritis was approved by the Alfred Hospital and Cabrini Medical Centre Human Research Ethics Committees. All women gave informed consent for participation.

Inclusion criteria for participation included: age over 45 years, postmenopausal status defined by the absence of menstrual periods for at least 12 months, a diagnosis of rheumatoid arthritis defined by 1987 American Rheumatology Association (ARA) criteria [16]. Exclusion criteria included: current or previous treatment with bone active agents other than hormone replacement therapy, calcium or vitamin D, history of malignancy, renal impairment where the serum creatinine was more than 0.2 mmol/l, and unable or unwilling to give informed consent. In Australia patients can choose to have specialist treatment in public (entirely government funded) or private (government subsidised) care settings. The 5 recruitment settings included two university affiliated metropolitan public hospital ambulatory care general rheumatology clinics and three specialist rheumatology private practices. Study participants were recruited in a consecutive fashion using manual medical record screening.

Disease assessment was performed by a trained metrologist. Markers of disease activity included: duration of morning stiffness (minutes), 68 swollen joint count, current erythrocyte sedimentation rate (ESR, mm/hr). A composite disease activity score, the DAS28T+S score [17] was calculated. Markers of disease severity included: disease duration (defined from the time of physician diagnosis), presence of rheumatoid factor, radiological appearance of typical rheumatoid arthritis erosions, numbers of disease modifying anti-rheumatic drugs (DMARDs), number of joint arthroplasty procedures, and presence of extra-articular features (nodules/vasculitis). Functional status was assessed using the Modified Health Assessment Questionnaire (MHAQ) [18] and the American College of Rheumatology global functional status [19]. A patient self-rated global health score was also collected (excellent, good, fair, poor). Prednisolone use was defined as 'current use' or 'ever used'. Grip strength was measured using a calibrated instrument and the average of three measurements on the dominant and non-dominant side were recorded. The following osteoporotic risk factors were assessed: age (years), body mass index (BMI) <20 kg/m^2 ^[weight in kilogram/(height in metres)^2^], current smoker, alcohol >3 standard glasses per day, caffeine >3 standard glasses per day, sunlight exposure < 20 minutes three times per week, calcium intake (<1000 mg/day, <250 mg/day), previous history of low trauma fracture.

Bone mineral density was assessed by DXA (Lunar or Hologic devices) at a geographical location defined by patient or clinician preference. This was designed to reflect normal clinical management. Osteopenia was defined as a T-score less than -1, and osteoporosis as a T-score less than or equal to -2.5 at the femoral neck [20]. There is no standardised quality assurance protocol, nor consistent use of a normative reference dataset between DXA machines which may lead to inter-site measurement error, especially when comparing mean BMD levels. For this reason, analysis of clinical decision tool performance was based on prediction for T scores at the femoral neck only.

### Clinical decision tools and Statistical analysis

The clinical decision tools are summarised in Table 1. They were applied using methodology described within original development and validation studies [5,6,15,21-24] to assess their predictive value in identifying bone mineral density scores of less than or equal to -2.5 at the femoral neck. The sensitivity, specificity, positive predictive and negative predictive values and area under the curve were assessed as a measure of combined sensitivity/specificity model behaviour [25]. The diagnostic attributes of the clinical decision tools were compared by examination of the area under the receiver-operator-curve (ROC). The tool with the highest area under curve was arbitrarily chosen as the comparison point using the "rocgold" command within Stata. (Stat 9.2, Copyright 1996–2007 StataCorp, College Station, TX 77845, USA).

**Table 1:** Summary of criteria described for clinical decision rules to predict low bone mineral density applied in a cohort of 127 post-menopausal women with rheumatoid arthritis.

**Guideline/Rule**	**Selection Cut Point**	**Scoring system**
Simple Calculated Osteoporosis Risk Estimation (SCORE)(6)	Score ≥ 6	Points for: Race: 5 if not black Rheumatoid arthritis: 4 if present Personal history of minimal trauma fracture after age 45 yrs years: 4 each fracture of the wrist, hip, or rib to a maximum of 12 Age: 3 times first digit of age in yrs Estrogen therapy: 1 if never used Weight: -1 times weight in lb divided by 10 and truncated to integer
Osteoporosis Risk Assessment Instrument (ORAI)(4)	Score ≥ 9	Points for: Age: 15 if 75 yrs or older, 9 if 65–74 yrs, 5 if 55–64 yrs Weight: 9 if <60 kg, 3 if 60.0–69.9 kg Estrogen use: 2 if not currently taking estrogen
Age, Body Size, No Estrogen (ABONE)(23)	Score ≥ 2	Points for: Age: 1 if > 65 yrs Weight: if <63.5 kg Estrogen use: 1 if never used oral contraceptives or estrogen therapy for at least 6 months
Body weight criterion(22)		If Weight <70 kg
Osteoporosis Self Assessment Tool (OST)(8)	Score < 2	Sum of: (Weight – Age) * 0.2 truncated to an integer
Study of Osteoporotic Fractures (SOFSURF)(7)	Score ≥ 1	Sum of: (Age – 65) * 0.2 Plus 1 point each for Weight less than 68 kg Current smoker Personal history of postmenopausal fracture Plus 2 points if Weight < 59 kg.
National Osteoporosis Foundation (NOF)(21)	Score ≥ 1	One point each for: Age ≥ 65 yrs Personal history of minimal trauma fracture >40 yrs Family history of fracture Current cigarette smoking
Amsterdam rheumatologists score(15)	Score ≥ 2	Points for: Disease activity (mean CPR > 20 mg/l or persistent ESR > 20 mm for the 1^st ^hour Age: women > 50 yrs Immobility: ARA score = 3 or HAQ score ≥ 1.25.
Modified Amsterdam(24)	Score ≥ 3	As per Amsterdam rheumatologist algorithm, with an additional point for each of Weight: <60 kg Any previous steroid use

## Results

Two hundred and fifty five women were screened, representing 62.5% total potentially eligible rheumatoid arthritis population. One hundred and thirty five women did not respond to invitations to participate. One hundred and twenty eight (50.2%) screened women were excluded due to current or previous use of a bone active medication (48, 37.5%), pre-menopausal status (47, 36.7%), medical co-morbidity (15, 11.7%) or refusal to participate (6, 4.7%). One hundred and twenty seven postmenopausal women consented to participate in the study. Seventy-two (57%) had no record of a previous DXA assessment. Sixteen (12.6%) women had a history of post-menopausal fracture.

The median age of participants was 62 yrs (interquartile range {IQR} 56–71). The majority (124, 98%) were Caucasian. Disease duration was long [median 108 months (IQR 60–168)]. Although only 10, (7.9%) reported a global health score of fair or poor, 72 (57%) were ARA functional status 111 and weakness to rise from a chair was documented in 39 (30.7%). Other parameters including arthroplasty (22, 17.3%), use of more than one disease modifying anti-rheumatic drug (33, 25.9%) and current use of prednisolone (44, 35%) are in keeping with a rheumatoid arthritis population of long disease duration. In contrast, evidence of active joint disease was less impressive with a low active joint count (median tender = 5, IQR = 2–10, median swelling = 5, IQR = 2–9,), and short duration of early morning stiffness (median = 30 minutes 5, IQR = 5–60 Patients with high disease activity (as measured by the DAS28T+S(17) had greater duration of early morning stiffness (Spearman rho = 0.663, p < 0.001).

There was a high prevalence of low dietary calcium intake (median 500 mg/day, IQR 250–825) and 15 (12%) had less than 250 mg/day. 34 (27%) of women had 3 or more risk factors for osteoporosis. Postmenopausal fragility fractures were documented in 16 (13.0%) women, 43 (33.9%) recalled a fall within the twelve months prior to assessment and for 17 (13.4%) this included two or more falls. Fear of falling was reported as moderate or severe in nearly one third of women.

The results of BMD are summarised in Table 2. Overall, 80 (64.5%) women had BMD T-scores less than -1, of whom 56 (45.2%) were less than -1 but greater than -2.5 and 24 (19.4%) were less than or equal to -2.5. There were no clear differences in fracture and non fracture groups (femoral neck mean t-score = -1.52, sd = 1.33 for fracture group, compared with -1.09, sd = 1.38 for non fracture group, p = 0.247, and lumbar spine mean t-score = -0.34 for fracture group compared with -1.09, sd = 1.38 for non-fracture group, p = 0.870). The degree of current disease activity (as measured by DAS28T+S) was not related to bone mineral density (Spearman rho for t-score at femoral neck = 0.049, p = 0.624, rho for t-score at lumber spine = -0.098, p = 0.323).

**Table 2:** Results of bone mineral density measured by DXA postmenopausal women with Rheumatoid Arthritis (n = 127).

Site	% T score > -1	T < -1 to -2.5 N (%)		T < -2.5 N (%)		Valid N
**Total Group N = 127**						

Femoral Neck	87.2	52	(42.3)	18	(14.6)	123
Lumbar Spine	90.5	45	(31.7)	12	(9.8)	123

**Non Fracture Group**						
Femoral Neck	88.2	40	(37.4)	14	(13.1)	107
Lumbar Spine	91.1	30	(28.0)	10	(9.3)	107

**Fracture group**						
Femoral Neck	80.0	9	(56.3)	4	(25.0)	16
Lumbar Spine	86.7	6	(37.5)	2	(12.5)	16

The performance attributes of the scoring algorithms are summarised in Table 3. None of the seven general scoring algorithms that were tested performed significantly better than the others as assessed by ROC curve analysis (range 0.63–0.76). The rheumatoid arthritis specific guideline had the lowest area under the curve. When compared to the OST, which had the highest area under ROC, the only tool that performed substantially poorer was the "low body weight" criterion (Bonferoni adjust p < 0.001).

**Table 3:** Attributes of clinical decision tools for predicting T Score less than or equal to -2.5 (at the femoral neck) in 127 post-menopausal women with rheumatoid arthritis

			**Predictive values**		
T-FN < -2.5	Sensitivity (95% CI)	Specificity (95% CI)	Positive (95% CI)	Negative (95% CI)	Area Under Curve (95% CI)
SCORE	100 (82–100)	10 (5–17)	16 (10–24)	100 (69–100)	0.73 (0.59–0.86)
ORAI	72 (47–90)	50 (40–60)	20 (11–31)	91 (81–97)	0.73 (0.59–0.87)
NOF	94 (73–100)	46 (36–56)	23 (14–34)	98 (89–100)	0.75 (0.65–0.85)
ABONE	56 (31–79)	84 (75–90)	37 (19–58)	92 (84–96)	0.72 (0.58–0.85)
OST	78 (52–94)	51 (41–61)	22 (12–34)	93 (83–98)	0.76 (0.64–0.89)
SOFSURF	89 (65–99)	34 (25–44)	19 (11–29)	95 (82–99)	0.72 (0.59–0.85)
Low Body Weight	72 (47–90)	53 (43–63)	21 (12–33)	92 (82–97)	0.63 (0.51–0.74)
Amsterdam	100 (82–100)	7 (3–14)	16 (10–24)	100 (59–100)	0.64 (0.51–0.76)
Modified Amsterdam	78 (52–94)	44 (34–54)	20 (11–31)	92 (80–98)	0.70 (0.57–0.83)

Patients from public hospital rheumatology clinics had more severe rheumatoid arthritis (poorer functional status, longer early morning stiffness and higher swollen joint count) and a higher ESR. Therefore, the diagnostic models were applied with and without the public patients included. The ROC values did not alter significantly therefore the reported values included all patients.

## Discussion

Our findings of a high prevalence of risk factors for osteoporosis and low BMD among a postmenopausal population of women with rheumatoid arthritis are in keeping with previous studies. Rheumatoid arthritis has been reported to be associated with localised [9] and generalised bone loss [26,27], increased risk of osteoporosis and low trauma fractures [10,28]. Factors associated with low bone density include disease activity, disability disease duration as well as therapy with corticosteroids [29-31].

Further, in this study we have demonstrated the limited utility of clinical decision tools, and lack of advantage of rheumatoid arthritis specific clinical decision tools for predicting osteoporosis in this population. This is also in keeping with previous studies of non selected post-menopausal populations. Lydick et al [6] developed and validated a questionnaire, the Simple Calculated Osteoporosis Risk Estimation ("SCORE") which included 6 variables (age, race, rheumatoid arthritis, non-traumatic fracture >45 years, oestrogen use, weight) and reported sensitivity of 89% and specificity of 50% for the tool. However, when this "SCORE" was applied by Cadarette et al [5] to a post-menopausal population in Toronto they found it to have very poor specificity, with a high false positive rate of 68%. More recently, application of three clinical decision tools (SCORE, ORAI, NOF) to an independent post menopausal population reported poor utility as a general screening method [32]. If algorithms that give a point estimate for sensitivity of at least 90% for prediction of osteoporosis are considered, the algorithms that approximate or exceed this criteria in our patient population without modification from the developers recommendations, were the SCORE (100%) [6], Amsterdam(100%) [15], NOF (94%) and SOFSURF(89%) [7]. However, the corresponding specificities are 10% and 7% for SCORE and Amsterdam respectively, suggesting it would be possible to exclude osteoporosis without a DXA study in only approximately 10% of patients who do not have the condition. The SOFSURF, with a somewhat lower sensitivity, would reduce the number unnecessary DXA studies by approximately 34%. In our study population the best performing tool was the NOF which, in addition to providing high sensitivity, has the potential to reduce the number of unnecessary DXA studies by approximately 45%.

Useful clinical decision tools need to be able to take account of individual risk factor burden, including reversible risk, and be easy to administer [33]. Poor predictive performance of the clinical decision tools in our patient population may be due to a number of factors. Firstly, there is well known heterogeneity of osteoporosis risk in different populations [34]. Secondly, heterogeneity within the rheumatoid arthritis population may contribute to poor predictive utility. A single clinical decision tool may not account for variability in osteoporosis risk that is influenced by disease duration, activity and severity [35]. Although we did not find differences in the performance of the clinical decision tools between public and private patients in whom some differences in disease activity and severity were noted, this study was not powered to demonstrate such differences and larger studies are required to assess this hypothesis. Overall, our data adds support to a recent systematic review of the performance of the Osteoporosis Self-Assessment Tool (OST) that suggests clinical decision tools may be more useful in identifying a subset of patients who are at low risk of osteoporosis and do ***not ***need formal bone mineral density assessment [36].

The rationale for using clinical decision tools to predict osteoporosis is that BMD is known to be a major risk factor for fracture and that there is a site specific gradient to risk [33]. Unless there is consensus about the long-term safety and effectiveness of fracture prevention therapy as well as demonstrated high levels of compliance with preventive therapy, applying clinical decision tools that accurately predict low risk of osteoporosis may support cost-effective approaches to diagnosis and management. However, previous reports, supported by our data, indicate that fracture can occur in the presence of normal BMD. In addition, other factors, independent of BMD can be important predictors of an individual's incident fracture risk [33]. Whilst one of the major independent predictive factors, age is not reversible, other factors including falls, reduced mobility and use of corticosteroids all of which are important to our patient population, may be amenable to non pharmacological interventions. Therefore, clinical indices for predicting fracture in addition to BMD measurement would potentially provide greater utility for clinical management [33]. The number of patients with fracture was too small in our population to test decision tool predictive performance for fracture rather than osteoporosis, however other authors report limited utility of clinical indices for prediction of fracture risk in postmenopausal populations [37]. This need to be further investigated for patients with rheumatoid arthritis and other high risk populations.

The study had further limitations. Bone densitometry was performed using a variety of different DXA machines that may have contributed to significant undetected technical error in measurement [33]. However, the study was specifically designed to provide a pragmatic clinical population assessment and it is unlikely that this would have significantly affected the results. Consecutive recruitment of patients was undertaken to reduce selection bias, however individual practitioner practice behaviours may have also influenced the utility of clinical decision tools. The study was not powered to assess this question. The study also was restricted to a female post-menopausal population and the results may not be generalisable to males with rheumatoid arthritis. Generalisation to a wider community rheumatoid arthritis population is limited as patients with mild rheumatoid arthritis, not regularly attending a rheumatologist, may have been excluded from assessment. Inclusion of such patients however would be more likely to reduce the utility of the tools.

Even excluding patients in whom there was current or previous treatment with bone active agents other than HRT, calcium or vitamin D, there were over 10% with a history of fracture since the age of 50 years. As the study did not include assessment of appropriateness of care, nor validate self reported information with general practice records and radiographs, it is not possible to draw conclusions from this data.

## Conclusion

In summary, rheumatoid arthritis specific clinical decision tools for identifying postmenopausal women with RA who have low bone mineral density and osteoporosis demonstrated no greater performance than clinical decision tools developed for use in non selected postmenopausal populations. A trade-off between clinical decision tools' high sensitivity performance and avoidance of unnecessary DXD scanning needs to be further investigated in a larger RA population study where the influence of clinician osteoporosis management, heterogeneity of RA disease factors and independent risk factors for fracture can be assessed.

## Competing interests

The author(s) declare that they have no competing interests.

## Authors' contributions

CB – contributed to study design, project management, data analysis design, interpretationand writing of the article.

SH – contributed to study design, patient recruitment, data interpretationand writing of the article.

AL – contributed to data analysis design, analysis and writing of the paper.

## Pre-publication history

The pre-publication history for this paper can be accessed here:


